# Retrospective detection of SARS-CoV-2 in hospitalized patients with influenza-like illness

**DOI:** 10.1080/22221751.2020.1785952

**Published:** 2020-07-05

**Authors:** Xiaoqi Yu, Dong Wei, Yongyan Chen, Donghua Zhang, Xinxin Zhang

**Affiliations:** Research Laboratory of Clinical Virology, Ruijin Hospital, and Ruijin Hospital North, Shanghai Jiao Tong University School of Medicine, Shanghai, People’s Republic of China

**Keywords:** SARS-CoV-2, COVID-19, epidemiology, prevalence, influenza

## Abstract

Since the first report of the coronavirus disease (COVID-19) in late December 2019, the pandemic caused by severe acute respiratory syndrome coronavirus 2 (SARS-CoV-2) has now widely spread to more than 187 countries and regions. However, it is unclear whether there has been cryptic transmission before these early officially confirmed cases, we therefore retrospectively screened for the SARS-CoV-2 RNA in 1271 nasopharyngeal swab samples, as well as the prevalence of IgM, IgG, and total antibodies against SARS-CoV-2 in 357 matched serum samples collected from hospitalized patients with influenza-like illness between 1 December 2018 and 31 March 2020 in Shanghai Ruijin Hospital. The onset date of the earliest COVID-19 case in this study was 25 January 2020. Before this time point, the presence of SARS-CoV-2 was not observed, which limited the possibility that SARS-CoV-2 has already spread among the population before the large-scale outbreak. Additionally, among 6662 patients with influenza-like illness from 1 December 2017 to 31 March 2020, the overall number of patients positive for influenza and other respiratory viruses during the COVID-19 period decreased significantly when compared with that in the same period of the last two years, reflecting that public health interventions can effectively control the spread of common respiratory viruses.

The COVID-19 outbreak was first reported in Wuhan, Hubei Province, China, and it has now spread rapidly worldwide [1]. As of 17 May 2020, a total of 4,650,793 COVID-19 cases have been confirmed, with 312,115 deaths. SARS-CoV-2 is the causative pathogen of COVID-19, identified as the seventh type of coronavirus to infect humans [2]. Six other kinds of coronaviruses are known to cause human diseases, including SARS-CoV and MERS-CoV with high mortality, whereas 229E, OC43, NL63, and HKU1 are associated with mild symptoms [3].

Wuhan is the largest city and is the most important transportation hub in central China. It is estimated that nearly 5 million residents left Wuhan before the city lockdown on 23 January 2020 [4]. Due to the large population movement during the Chinese Spring Festival, person-to-person transmission caused the infection spread throughout the country. Lack of rapid molecular diagnostic assays at that time also made it difficult to monitor the chains of transmission in the early stage of the pandemic. On 20 January 2020, the National Health Commission confirmed the first imported COVID-19 case in Shanghai. The time of the large-scale outbreak coincided with seasonal influenza and other respiratory illnesses, and the clinical symptomatology of COVID-19 is similar to that of influenza-like illness (ILI) [5], including fever, cough, and sore throat [6]. To verify whether there was unrecognized transmission before the outbreak, we aim to detect the presence of SARS-CoV-2 among 1271 hospitalized patients with ILI in our hospital between 1 December 2018 and 31 March 2020.

The patients included in this study comprised 796 males and 475 females, and the median age was 66 years (IQR, 53-82). Considering the reliability of the real-time PCR assay for SARS-CoV-2 RNA detection, we confirmed the result using two different commercial kits to avoid any false positive or negative results. Overall, three patients were positive for SARS-CoV-2. The onset date of the earliest case was 25 January 2020 in this study, five days after the first reported case in Shanghai. The date of the other two positive samples was 27 January 2020 and 16 March 2020, respectively.

Additionally, we detected the prevalence of specific antibodies against SARS-CoV-2 in 357 matched serum samples collected from hospitalized patients with ILI. Serological tests can prevent false negative results caused by the inappropriate timing of sample collection and poor sample quality in nasopharyngeal swabs [7]. The results of previous studies confirmed that IgG antibody could persist beyond 7 weeks, and the profile of specific antibodies against SARS-CoV-2 is similar to that against SARS-CoV [8]. The production of IgM antibody was positive as early as day 1 after symptom onset, and this would be helpful for the early diagnosis of COVID-19 patients [9]. In this study, three patients with positive SARS-CoV-2 RNA also tested positive for IgM antibody, while negative for IgG antibody, all the remaining patients showed negative results for IgM, IgG, and total antibodies, indicating that there was no previous exposure to SARS-CoV-2 during that time period.

We further compared the epidemiological profiles of influenza and other respiratory viruses during the COVID-19 period with those of the same period of the last two years. There were 678 cases from 1 December 2017 to 31 March 2018, 1023 cases from 1 December 2018 to 31 March 2019, and 1390 cases from 1 December 2019 to 31 March 2020. Interestingly, the positive rate for influenza and other respiratory viruses was 6.90% during the COVID-19 period, which was relatively low compared with that of 18.88% and 24.05% in the last two years. Among all 3091 samples, 470 (15.21%) were tested positive for a variety of different viruses (Figure 1(A)), and the proportion of sample testing positive for different viruses has not changed significantly among different years. Influenza virus was the most common respiratory virus (8.44%), followed by respiratory syncytial virus (3.05%), rhinovirus (1.93%), and human metapneumovirus (1.31%), etc. The winter peak of seasonal influenza has occurred in all three years, but there was also a sudden drop in February 2020 (Figure 1(B)). These phenomena were largely due to the effective containment efforts of the Chinese government and the awareness of isolation and protection among the population, which block the transmission of respiratory viruses.

**Figure 1.**
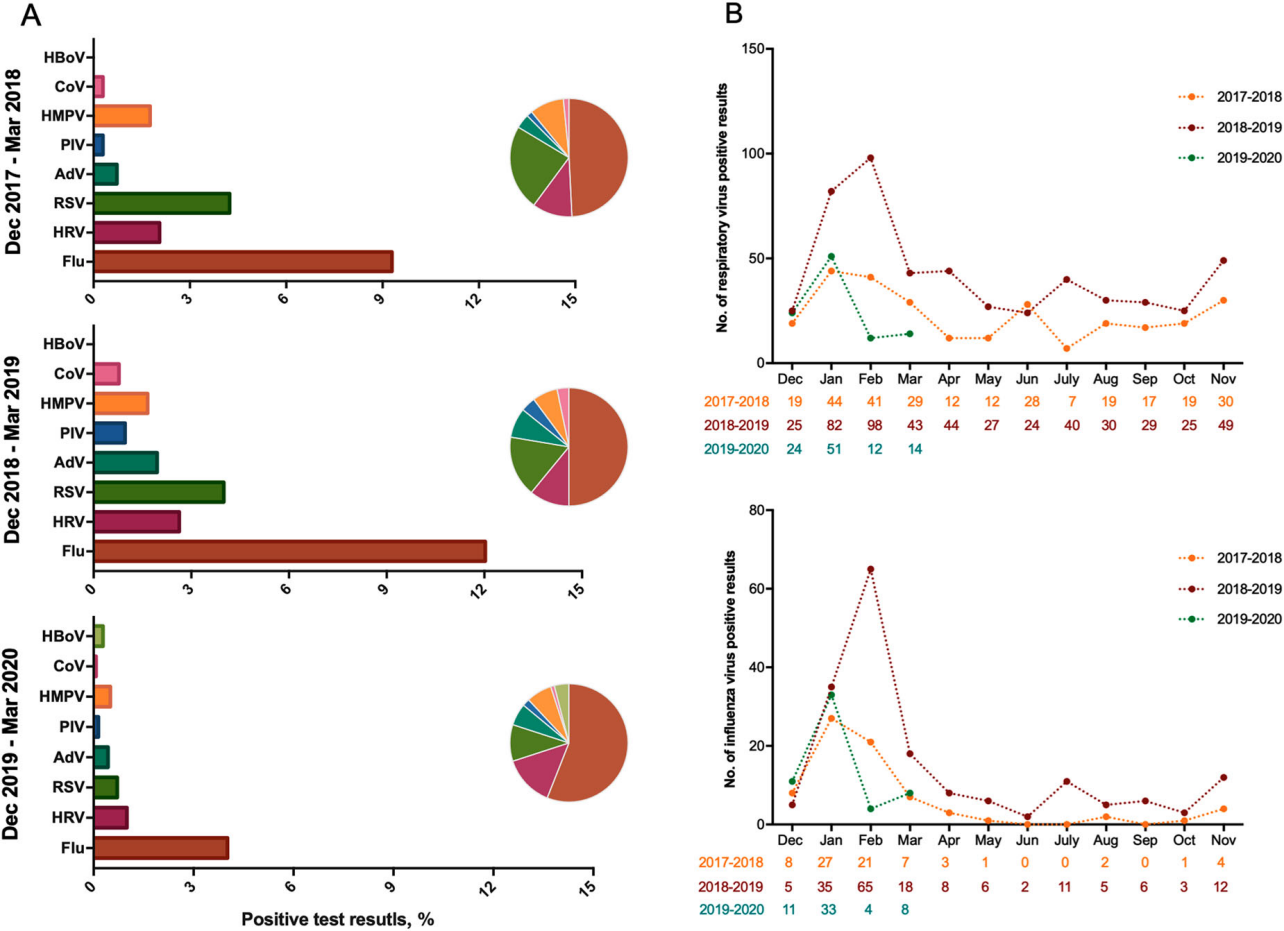
The epidemiological profiles of respiratory viruses. (A) The positive rate and proportion of different respiratory viruses identified from 3091 hospitalized patients with influenza-like illness during the COVID-19 period and the same period of the last two years. (B) All detected respiratory viruses and influenza virus in a total of 6662 hospitalized patients with influenza-like illness from Dec 2017 to Mar 2020. Numbers of positive results at each time point are given below.

Identifying the early epidemiological characteristics of a newly emerging infectious disease is of great interest, which helps us better understand the transmission chain and determine the origin of the virus. Thus far, the proximal origin of SARS-CoV-2 remains unclear. A bat coronavirus RaTG13 is highly similar to SARS-CoV-2 [10], with an overall identity of 96.2% at the whole genome level, though the receptor-binding domain (RBD) of the S gene (the most variable region) was highly divergent. Whereas the RBD of a coronavirus isolated from a Malayan pangolin has only one amino acid different from that of SARS-CoV-2, and the comparative genomic analysis suggested that SARS-CoV-2 might have originated from the recombination of Pangolin-CoV with RaTG13 [11]. It is generally accepted that bat is the natural reservoir host of SARS-CoV-2; however, the exact intermediated animal host in the process of transmission that enables the precursor virus of SARS-CoV-2 to evolve through natural selection remains uncertain.

Thus far, all SARS-CoV-2 genomes sequenced shared a similar genomic feature; hence, it is speculated that there might be ancestors of SARS-CoV-2 among the population, and the adaption process has occurred during undetected person-to-person transmission before identification of the early COVID-19 cases [12]. In this study, we did not detect the presence of SARS-CoV-2, and the negative qPCR results of the conserved regions in ORF1ab, N gene, and E gene indicated that the potential highly homologous progenitor virus of SARS-CoV-2 is less likely to be present in these patients, which reduces the possibility of prior exposure to SARS-CoV-like coronaviruses in the population before the outbreak, and means natural selection is more likely to occur in the animal host before zoonotic transfer.

The main concerns of the screening results were the relatively small sample size and sampling that is limited to a single hospital. Shanghai Ruijin Hospital is a public hospital, receiving patients from all over China, so these samples are representative of the general population to some extent. It should also be noted that after the COVID-19 outbreak, Shanghai has established a strict surveillance system, and most of the highly suspected cases were directly sampled and detected by Shanghai Municipal Center for Disease Control & Prevention (SCDC). In addition to the three mentioned cases with nasopharyngeal swab samples, a total of nine suspected cases were found in Shanghai Ruijin Hospital, and they were all transferred to the Shanghai Public Health Clinical Center after diagnosis.

The epidemiological profiles of respiratory viruses could be influenced by both weather conditions and indoor crowding. Since the COVID-19 outbreak, a series of public health interventions was taken, including cordons sanitaire, traffic restriction, social distancing, home quarantine, centralized quarantine, and universal symptom survey [13]. These interventions led to the decrease in SARS-CoV-2 infected cases and were also associated with improved control to infection by influenza and other respiratory viruses, which suggested that prevention measures can have a large public health impact.

In summary, our work provided additional information to the unknown early spread of SARS-CoV-2 before the outbreak of the current pandemic. Nevertheless, pathogenic and serological investigations are still needed for the systematic population of different regions to confirm whether there was such cryptical spread, which could replenish the history of the COVID-19 transmission.

## Materials and methods

### Sample collection

The nasopharyngeal swab samples for this study were collected from 1271 hospitalized patients with influenza-like illness from 1 December 2018 to 31 March 2020 in Ruijin Hospital (Shanghai, China). Matched serum samples were also collected from 315 patients. Nasopharyngeal swab samples were preserved in universal transport medium, and all samples were stored at −80°C until performing the related tests. This study was approved by the Ethics Committee of Ruijin Hospital.

### Detection of SARS-CoV-2, influenza and other respiratory viruses

Viral RNA was extracted from nasopharyngeal swab samples using the QIAamp Viral RNA Mini Kit (Qiagen), according to the manufacturer’s instructions. Real-time polymerase chain reaction (PCR) assay was performed for detecting the presence of SARS-CoV-2 using commercial kits (BGI, China; Life River, Shanghai, China) with the permission from the China Food and Drug Administration (CFDA). Influenza (Flu) and other respiratory viruses, including respiratory syncytial virus (RSV), parainfluenza virus (PIV), rhinovirus (HRV), human metapneumovirus (HMPV), enterovirus (EV), adenovirus (AdV), bocavirus (HBoV), coronavirus (OC43, 229E, and HKU1), severe acute respiratory syndrome coronavirus (SARS-CoV) were detected by real-time PCR assay (Roche, Germany). The detection procedures were performed by three trained technicians who were blinded to the sample’s information.

### Serological measurement

The specific IgM and total antibodies against SARS-CoV-2 in serum samples were detected using a chemiluminescent immunoassay (CLIA), which targets the receptor-binding domain (RBD) of the spike protein. (Beijing Wantai Biological Pharmacy Enterprise Co., Ltd). The specific IgG antibody was detected using colloidal gold-immunochromatographic assay (LIVZON, Zhuhai, China). Both commercial kits used were certificated and approved by the China Food and Drug Administration (CFDA), and were used at the same time to ensure the accuracy of serological measurement.
